# On the Calculation of Sample Entropy Using Continuous and Discrete Human Gait Data

**DOI:** 10.3390/e20100764

**Published:** 2018-10-05

**Authors:** John D. McCamley, William Denton, Andrew Arnold, Peter C. Raffalt, Jennifer M. Yentes

**Affiliations:** 1 MORE Foundation, 18444 N 25th Ave., Suite 110, Phoenix, AZ 85023, USA; 2 Center for Research in Human Movement Variability, Department of Biomechanics, University of Nebraska at Omaha, 6160 University Drive South, Omaha, NE 68182-0860, USA; 3 Julius Wolff Institute for Biomechanics and Musculoskeletal Regeneration, Charité-Universitätsmedizin Berlin, Augustenburger Platz 1, 13353 Berlin, Germany; 4 Department of Biomedical Sciences, University of Copenhagen, Blegdamsvej 3B, Copenhagen N 2200, Denmark

**Keywords:** range of motion, joint angle, gait, complexity, regularity

## Abstract

Sample entropy (SE) has relative consistency using biologically-derived, discrete data >500 data points. For certain populations, collecting this quantity is not feasible and continuous data has been used. The effect of using continuous versus discrete data on SE is unknown, nor are the relative effects of sampling rate and input parameters *m* (comparison vector length) and *r* (tolerance). Eleven subjects walked for 10-minutes and continuous joint angles (480 Hz) were calculated for each lower-extremity joint. Data were downsampled (240, 120, 60 Hz) and discrete range-of-motion was calculated. SE was quantified for angles and range-of-motion at all sampling rates and multiple combinations of parameters. A differential relationship between joints was observed between range-of-motion and joint angles. Range-of-motion SE showed no difference; whereas, joint angle SE significantly decreased from ankle to knee to hip. To confirm findings from biological data, continuous signals with manipulations to frequency, amplitude, and both were generated and underwent similar analysis to the biological data. In general, changes to *m*, *r*, and sampling rate had a greater effect on continuous compared to discrete data. Discrete data was robust to sampling rate and *m*. It is recommended that different data types not be compared and discrete data be used for SE.

## 1. Introduction

Measures of entropy have become increasingly popular as a means to describe the predictability of a signal. Approximate entropy was introduced as a measure of regularity useful in differentiating periodic, deterministic, and chaotic signals [1]. This measure was originally developed on QRS intervals of heart rate data from the electrocardiogram [2] and has since been widely used for the assessment of heart rate variability [3,4,5,6,7,8,9]. Subsequent applications on discrete data types have included hormone secretion rate [10], respiration rate [11], stride-to-stride minimum toe clearance [12,13], and stride and step time [14,15,16,17]. The application of entropy measures to human gait data (e.g., joint angles and stride characteristics) provides valuable information of the movement dynamics executed by the human motor control system [18,19].

It has been noted that the calculation of approximate entropy has a statistical bias [20], and sample entropy [21] was introduced to overcome this bias. Since its introduction, sample entropy has become a popular measure to describe the regularity of human data [14,15]. Sample entropy is a measure of the likelihood that vectors of length *m*, which are similar in a time series, remain similar for length *m* +1. Sample entropy has been found to be robust in the presence of observational noise [14,20]. Yentes et al. [14] concluded that 2000 points of theoretical data are necessary for a stable calculation of sample entropy values. Using biological data, sample entropy appeared to be stable under discrete data lengths from 500 to 5000 data points with most combinations of *m* and *r* [15]. To calculate sample entropy for 2000 stride intervals would require walking data to be recorded for approximately 33 min for a person walking at 60 strides per minute. These data collection times are generally not feasible, and for many less healthy subjects they are impossible to achieve. Consider a person with impaired or slower walking, collecting the minimum of 500 strides would require 15 minutes of continuous walking at a pace of 45 strides per minute. For this reason sample entropy has been calculated using a smaller number of data points or, in some cases, using continuous data [22,23,24,25,26].

Continuous data may be appropriate in cases where the regularity of the patterns within the interval of movement, e.g., within a stride, is of interest. Various forms of continuous data including joint angles [22,23,24,27] and trunk accelerations [25,26] have been used for the calculation of sample entropy. A simple understanding of the manner in which sample entropy is calculated would suggest that very small values of entropy would result from continuous, cyclical data. This is due to repetitive vectors of consecutive points being counted as similar because they fall within a radius, determined from the standard deviation calculated from the complete range of signal values. As the sampling rate is increased the interval between consecutive points will be reduced accordingly, resulting in a further reduction in calculated sample entropy values. The depressive effect sampling frequency has on sample entropy has been noted previously [27,28,29]. Another alternative method of calculating sample entropy has been to downsample the continuous data by introducing a lag value into the calculation to remove correlations unrelated to the dynamics of the system [30]. It is unknown if comparisons between groups, conditions, or body segments using continuous data will result in similar findings to discrete data types.

For both discrete and continuous data, it might be expected that sample rate will have an influence on the calculated value [27,31,32]; however, if the effect is consistent then comparisons between groups might be performed as long as the data are collected in the same manner. Using peaks, or the timing of any recognizable point in cyclical data, to define intervals will be affected by the sampling rate of the data. The intervals will fall into bins which are integral multiples of the sampling interval. This binning effect will increase as the sampling rate is reduced and there are relatively fewer bins for interval values to fall into.

Due to a lack of consistency in findings within the body of literature examining entropy during human movement [14,33,34], it is imperative to determine the best methodologies for data collection and thereby, results reporting. If the lack of consistency is due to the types of data being utilized, this is an important factor that must be considered when reporting results. Further, if methodological or parameter combination differences contribute to the lack of consistency within reported results, this must also be considered. Therefore, the purpose of this paper was to determine if comparison of sample entropy values between body segments (ankle v. knee v. hip) using continuous data would provide similar results to comparisons of sample entropy values calculated for discrete data extracted from the same continuous data. Further, we aimed to understand if these comparisons are affected by the sampling rate and the input parameters of *m* and *r*. For this purpose, biological data were collected from subjects walking on a treadmill. To confirm findings from biological data, and to provide an understanding of how sample entropy is affected by changing sampling frequency and input parameters for known theoretical data, mathematically generated signals were analyzed as well. These generated signals were controlled for periodic, chaotic, and random changes in amplitude and period length. With aging and pathology gait patterns become more random [35], making it important to understand how the sample entropy algorithm is biased when applied to data with changes to the structure or underlying dynamics.

## 2. Materials and Methods

### 2.1. Biological Data

Eleven healthy young adult subjects (8M, 3F; age: 24 ± 3 years; height: 1.74 ± 0.7 m; weight: 75 ± 12 kg) were recruited to participate. All subjects provided informed consent as approved by the local Institutional Review Board. Subjects were required to have no injury nor related surgeries within the last year, no cardiac disease, and no musculoskeletal or neurological abnormalities that would alter gait.

Subjects wore a tight-fitting suit during data collection. Retroreflective markers were positioned on the sacrum and bilaterally on the anterior superior iliac spines, posterior superior iliac spines, greater trochanters, midthighs, lower front thighs, lateral knees, tibial tubercles, lower lateral shanks, lateral ankles, top of the second metatarsophalangeal joints, posterior heels, lateral fifth metatarsophalangeal joints, and lateral calcanei. Subject’s self-selected treadmill walking speed was determined using a previously described method [14]. After a minimum of five minutes of rest, subjects walked for 10 min at their self-selected speed on the treadmill (AMTI, Watertown, MA, USA). Marker trajectories in 3D space with a measurement error of less than 1mm, were recorded at 480 Hz using a 12-camera motion capture system (Motion Analysis Corp., Santa Rosa, CA).

**Figure 1:**
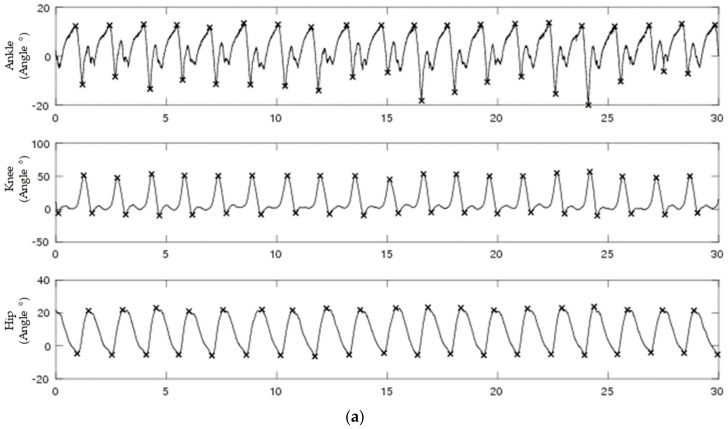

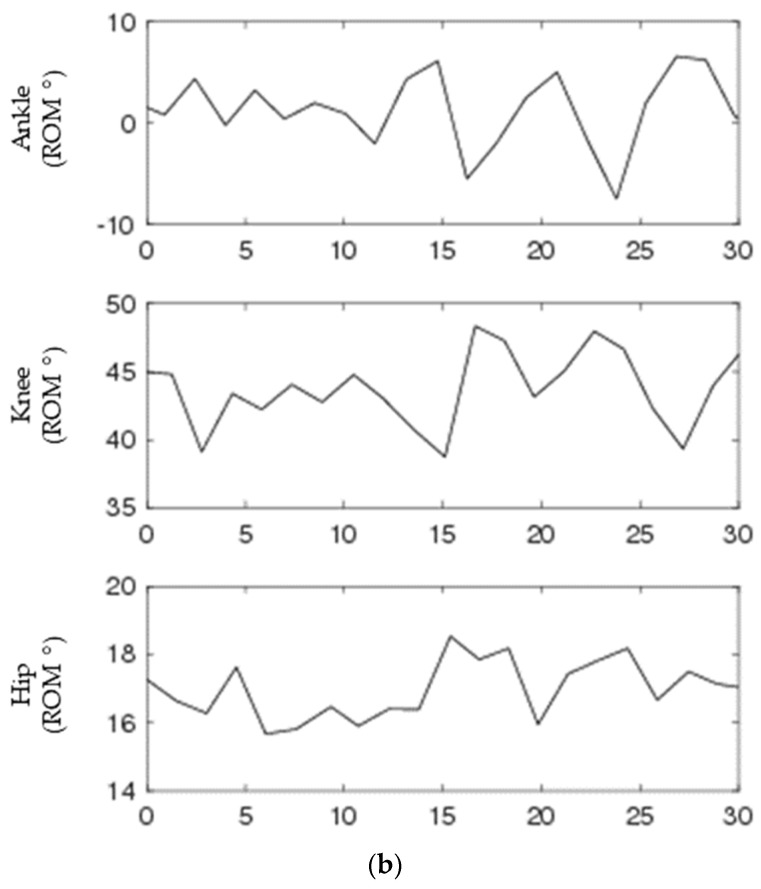
A 30-second segment of biological data for continuous (**a**) and discrete (**b**) for ankle (top), knee (middle), and hip (bottom) joint angles. Discrete signals were generated by subtracting peaks from valleys from each continuous joint angle.

Continuous sagittal plane ankle, knee, and hip joint angles of the right leg were calculated using Visual3D (C-Motion, Germantown, MD, Montgomery) (Figure 1). The trajectories of markers on the pelvis, thigh, shank, and foot were used to determine the instantaneous position and orientation of each of these segments in 3D space throughout each trial [36]. From these known orientations, the angles between each adjacent segment were calculated using the Cardan convention [37]. Tracking and calculating joint angles using markers has been shown to have errors of less than 4° for the knee and 3.5° for the ankle when compared to gold standard intracortical bone pins [38]. From the continuous joint angles, discrete range of motion time series were calculated as the difference between the maximum and minimum joint angle between heel strikes of the same foot using a custom MATLAB code (Mathworks, Inc., Natick, MA, USA).

### 2.2. Generated Data

Biological signals may fluctuate in multiple ways. Signals recorded for human walking may change due to altered step length (amplitude) or altered step time (period), or more commonly a combination of both. To understand the ability of sample entropy to differentiate between signals of varying regularity it is important to assess differences calculated from signals with known relative levels of regularity. For this purpose signals with periodic, chaotic, and random manipulations were generated. In total, nine different types of signals were generated. 

**Figure 2:**
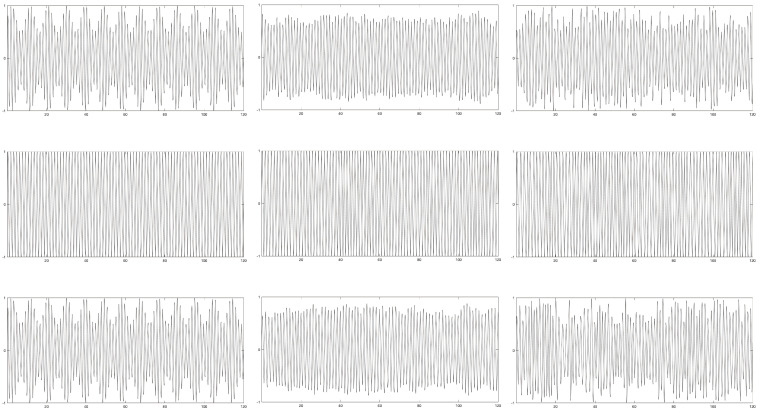
A 120-second segment of generated continuous data for periodic (**left**), chaotic (**middle**), and random (**bottom**) for amplitude (top), period (middle), and period + amplitude (bottom) manipulations. Discrete signals (Figure 3) were generated by subtracting peaks from valleys as denoted by x’s on each graph.

First, a sine wave was generated using MATLAB ® codes according to a 3 × 3 matrix (Supplementary materials). Manipulations consisted of (1) periodic, (2) chaotic, and (3) random manipulations to the sinusoids’ (1) cycle amplitude, (2) cycle period, or (3) both cycle amplitude and period. Thus, each sine wave had periodic, chaotic, or random manipulations to the magnitude, timing, or both magnitude and timing of the cycles within the wave (Figure 2). Periodic manipulations were made using sine function in MATLAB ®. Chaotic manipulations were generated using a pink noise generator. Random manipulations were generated using the ‘randi’ function in MATLAB ®. Individual continuous time series were generated to mimic the number of cycles of each subject as well as the time series’ data length. Meaning, a specific generated time series was generated to reflect each subject (e.g., if one subject had 500 cycles and another had 550, one set of generated files would have 500 cycles and the other would have 550).

Periodic manipulations to the sinusoids’ cycle amplitude had amplitudes that fluctuated sinusoidally between ±0.5 and 1. The cycle period was not altered. Chaotic manipulations to cycle amplitude generated sinusoidal cycles with amplitudes that fluctuated chaotically between ±0.5 and 1. Chaotic manipulations to cycle period also used the pink noise generator to alter the period of each cycle of the sine wave. Random manipulations to cycle amplitude generated sinusoidal cycles with pseudo random amplitude fluctuations between ±0.5 and 1. Random manipulations to cycle period altered the period between each cycle of the sine wave. A 10-point moving average filter was used on the signals with a random or chaotic cycle period to filter a ‘kink’ caused by the changing period between cycles. From the continuous sine waves, discrete time series were calculated as the between the peak and valley of each cycle using MATLAB ®.

### 2.3. Data Processing

**Figure 4:**
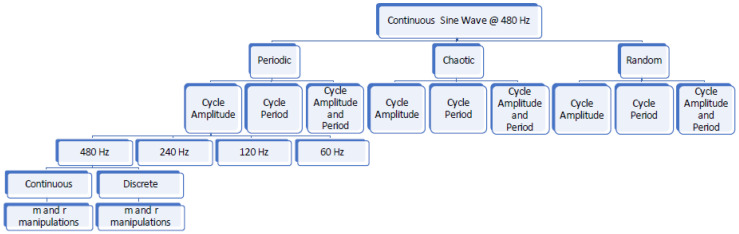
Flowchart showing data types generated and manipulations performed on these data. The steps followed in down sampling and extracting data series for calculation of sample entropy are only shown for the periodic, amplitude signal at 480 Hz. The same methods were applied to all other signals as well.

All data were inspected visually to ensure proper tracking and confirm that no outliers were present. Data length, N, of continuous data was 288,000 at 480 Hz data for 10 min of walking. All continuous time series were downsampled by factors of 2, 4, and 8 to represent sampling frequencies of 240, 120, and 60 Hz. Discrete time series were generated from the continuous time series at each of the sampling frequencies (480, 240, 120, and 60 Hz) per the methods above (Figure 4). Data lengths for each of the discrete time series were 566 ± 204 data points.

To calculate sample entropy Richman and Moorman [21] defined $B_{i}^{m}\left(r\right)$ as (*N* – *m* −1) times the number of vectors (of length m), X*_m_*(*j*) within r of X*_m_*(*i*) where j equals 1 to (*N* − *m*) and *j*≠*i*. Similarly, they defined A*_i_^m^* as (*N* – *m* −1) times the number of vectors (of length *m* + 1) X*_m_*_+1_(*j*) within r of X*_m_*_+1_(*i*) where *j* equals 1 to N-m and *j*≠*i*. From these values:

$$B^{m}\left(r\right)=\frac{\left(\sum_{j=1}^{N-m}B_{i}^{m}\left(r\right)\right)}{N-m}$$

and

$$A^{m}\left(r\right)=\frac{\left(\sum_{j=1}^{N-m}A_{i}^{m}\left(r\right)\right)}{N-m}$$

From which sample entropy was expressed as:

$$SE\left(m,r,N\right)=\;-\ln\frac{A^{m}\left(r\right)}{B^{m}\left(r\right)}$$

Sample entropy will equal zero when $A^{m}\left(r\right)$ = $B^{m}\left(r\right)$. Sample entropy is not defined when conditional probability $A^{m}\left(r\right)$ or $B^{m}\left(r\right)=0$. The smallest value of conditional probability that can be calculated is 1/2[(*N* − *m*)(*N* – *m* − 1)] which leads to a maximum value for sample entropy equal to ln(*N* − *m*) + ln(*N* – *m* − 1) − ln(2). Using the two different data types, sample entropy was calculated, using the methods described by Richman and Moorman [21] and Govindan et al. [30], for all signals using *m* = 2 and 3, and *r* = 0.10, 0.15, 0.20, 0.25, and 0.30 times the standard deviation of the signal, for a total of 40 different parameter combinations (two *m*-values, five *r*-values, and four sampling rates). Sample entropy was calculated on the continuous, 10-minute joint angles and generated counterparts using lags as defined by Govindan et al. [30]. This method discards correlations not related to the underlying dynamics of the system by introducing a lag, δ, between the vectors values. The value of δ is chosen as the point when the autocorrelation function falls below 1/*e*. The lags used for calculation of sample entropy for the continuous data are reported in Table 1. 

In addition, sample entropy was calculated on the biological and generated discrete time series. However, in some cases, typically when *r* = 0.10, the sample entropy value that was returned converged toward infinity (indicated in MATLAB ® files as a value of Inf), indicating that no matches were found for vectors of length *m* + 1. As these were strong outliers, they were removed from the data sets. For the range of motion data, nine were removed at the ankle, 13 at the knee, and 14 at the hip. For the discrete, generated data, between 3 to 20 values were removed from the following four combinations: (1) periodic manipulations to cycle period; (2) chaotic manipulations to cycle period; (3) periodic manipulations to cycle period plus amplitude; and (4) chaotic manipulations to cycle period plus amplitude.

### 2.4. Statistical Analysis

All data were checked for normality using a Shapiro–Wilk test. Ninety-nine percent of all biological data were normally distributed. All continuous, generated data were found to be not normally distributed. Discrete, generated data were normally distributed with the exception of two manipulations. Non-normally distributed data were transformed using a reciprocal approach. After transformation, all residuals demonstrated normal distribution. Three-way, repeated measure ANOVAs were used to compare the effect of sampling rate (Hz) and parameter settings (*m* and *r*) on the calculated sample entropy. Sample entropy values for continuous data were analyzed separately to sample entropy values for discrete data. Uncorrected Fischer’s least significant difference post-hoc tests were used. Significance was set at α = 0.05. Statistical analysis was conducted in SPSS (IBM, Armonk, NY, USA) and Prism7 (GraphPad Software, La Jolla, CA, USA).

## 3. Results

### 3.1. Joint Angle Data

Significant differences in regularity were found between almost every combination of sample rate, *m*, and *r* for the (1) ankle and knee; (2) ankle and hip; and (3) knee and hip. Regularity increased from the ankle to the knee and from the knee to the hip. The only exception was between the ankle and knee when *m* = 3 and *r* = 0.30 at all four sampling rates; no significant differences were found in these cases. Furthermore, at each joint, joint angles demonstrated significant main effects and interactions for sampling rate, *m*, and *r* (Table 2).

**Figure 5:**
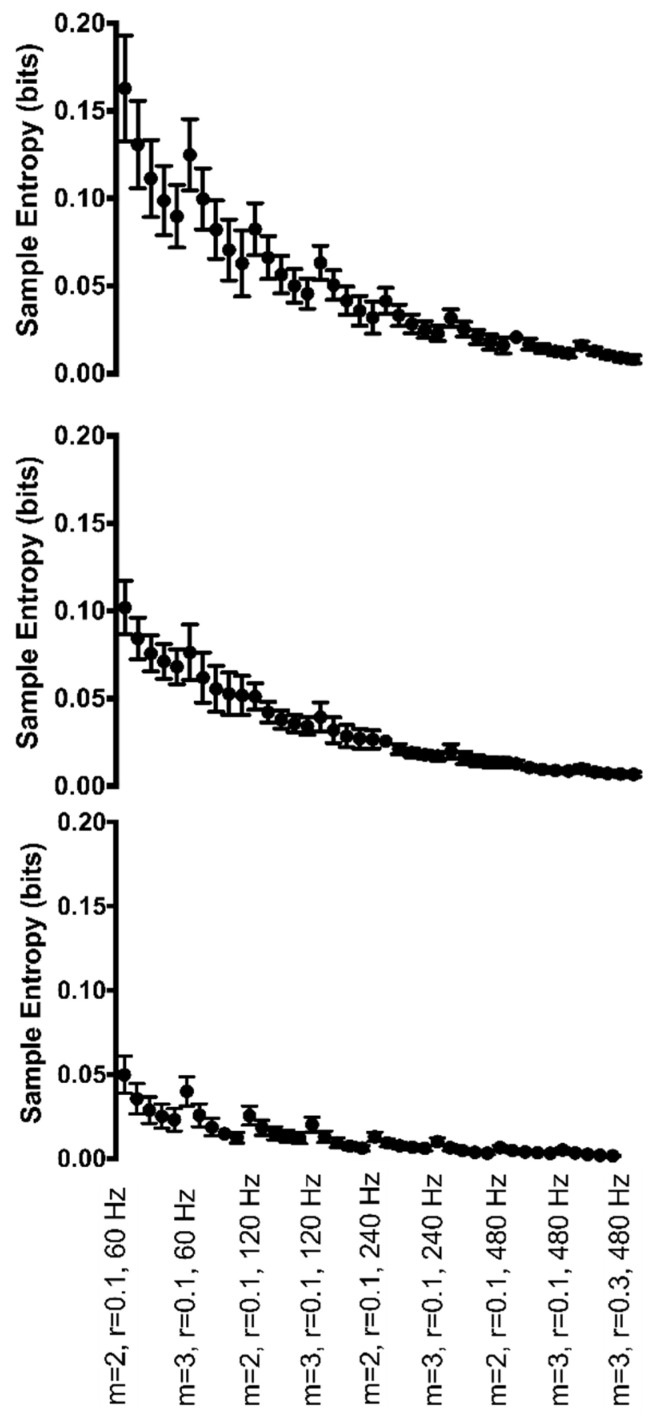
Group mean sample entropy of ankle joint angle (top row), knee joint angle (middle row), and hip joint angle (bottom row) for each Hz*r*m parameter combination. Sample entropy values are displayed on the y-axis. All parameter combinations are plotted along the x-axis starting with *m* = 2, *r* = 0.10, and 60 Hz at the left and moving to *m* = 3, *r* = 0.30, and 480 Hz at the right. For clarity, only the combination with *r* = 0.10 is labeled for each combination of *m* and sampling rate, with the *r* value increasing with each data point within each combination.

At the ankle, the interaction of Hz*r*m was significant (F(12,120) = 6.9, *p* < 0.001) (Figure 5, top row). Greater irregularity was found when *m* = 2 as compared to when *m* = 3. In general, regularity increased as the sampling rate increased. Further, regularity increased significantly as *r* values increased.

The knee joint angle revealed a significant 3-way interaction between Hz*r*m (F(12,120) = 8.9, *p* < 0.001) (Figure 5, middle row). Irregularity was greater when *m* = 2 than when *m* = 3. Regularity increased significantly as sampling rate decreased or as *r* values increased. It was found that 674 of the possible 780 pairwise comparisons were significant. 

At the hip, the joint angle had a significant interaction of Hz*m (F(3,30) = 31.0, *p* < 0.001) as well as a significant main effect of *r* (F(4,40) = 188.9, *p* < 0.001). Irregularity was greater when *m* = 2 than when *m* = 3. Regularity increased significantly as sampling frequency decreased or as *r* values increased. However, as the sampling rate decreased from 480 to 60 Hz, the effect of increasing *r* did not change the entropy value as drastically (Figure 5). Six-hundred-and-forty-two of the 780 possible pairwise comparisons were significant.

### 3.2. Biological Range of Motion Data

No significant difference in range of motion regularity was found between (1) ankle and knee; (2) ankle and hip; and (3) knee and hip. This was true for every combination of sample rate, *m*, and *r*. Moreover, at each joint, range of motion demonstrated a significant main effect of *r* (Table 2). 

**Figure 6:**
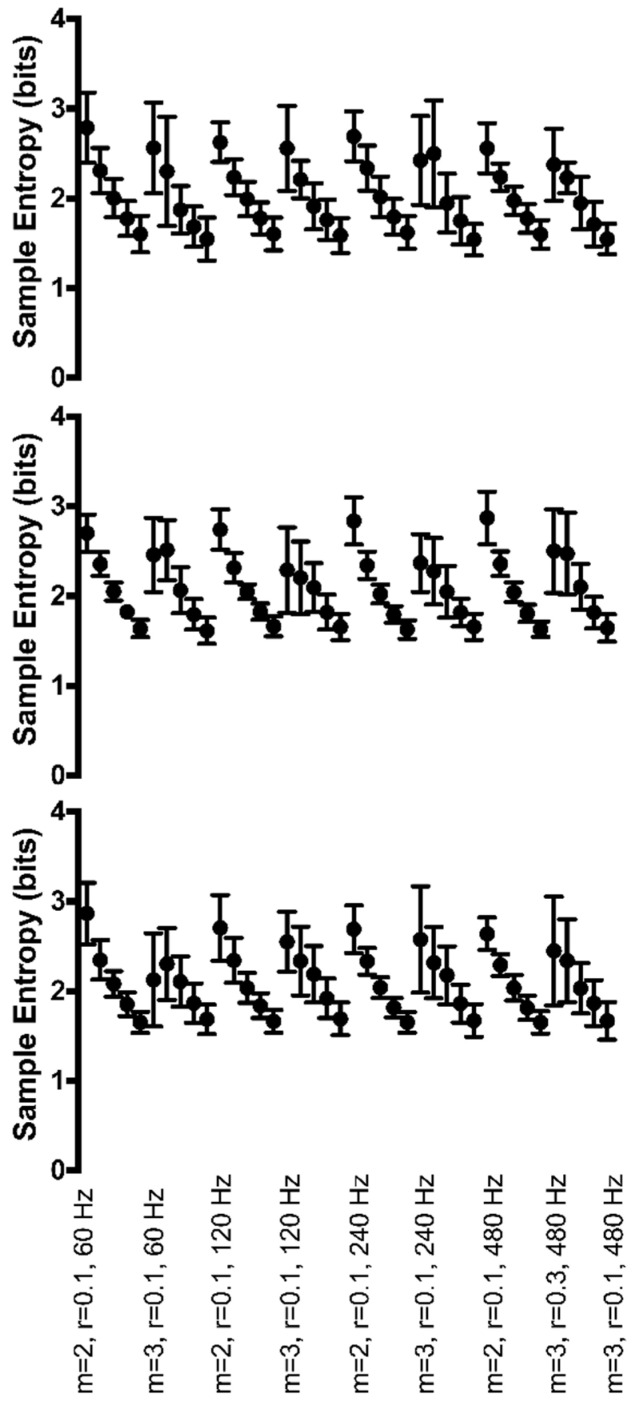
Group mean sample entropy of range of motion at the ankle (top row), knee (middle row), and hip (bottom row) for each Hz*r*m parameter combination. Sample entropy values are displayed on the y-axis. All parameter combinations are plotted along the x-axis starting with *m* = 2, *r* = 0.10, and 60 Hz at the left and moving to *m* = 3, *r* = 0.30, and 480 Hz at the right. For clarity, only the combination with *r* = 0.10 is labeled for each combination of *m* and sampling rate except the final combination.

At the ankle, a main effect of *r* was found to be significant (F(4,16) = 136.9, *p* < 0.001) for the range of motion time series (Figure 6, top row). As the *r* value increased from 0.10 to 0.30, irregularity decreased (sample entropy became smaller in value). No other significant interactions or main effects were found at the ankle (Figure 6).

The range of motion at the knee was found to have a significant interaction between Hz*r (F(12,24) = 2.3, *p* = 0.037) (Figure 6, middle row). Irregularity decreased as the *r* value increased. The sampling rate also influenced irregularity. The 60 Hz sample rate was the least consistent across *r* values. Regularity appeared to be least influenced at the 480 Hz sample rate when *r* was set greater or equal to 0.15. For the majority of *r* combinations, 120 Hz provided the greatest irregularity in knee range of motion.

Hip range of motion was found to have a significant 3-way interaction between Hz*r*m (F(12,36) = 4.7, *p* < 0.001) (Figure 6, bottom row). When *m* increased from 2 to 3, irregularity at 240 and 480 Hz increased; however, irregularity decreased at 60 Hz and 120 Hz with 60 Hz being the most affected by the increase in *m*. Regularity increased as *r* values increased across all sampling rates. Both *m* of 2 and 3 provided very similar regularity across four of the five *r* values. Only when *r* was very small, *r* = 0.10, was irregularity greater at *m* = 2 as compared to *m* = 3.

### 3.3. Continuous, Generated Data

The F- and *p*-values for the main effects of sample rate, *m*, and *r* and all interactions for the continuous, generated data are summarized in Table 3. Periodic manipulations had the greatest effect on cycle amplitude; whereas, chaotic and random manipulations had the greatest effect on cycle period.

#### 3.3.1. Periodic Manipulations

**Figure 7:**
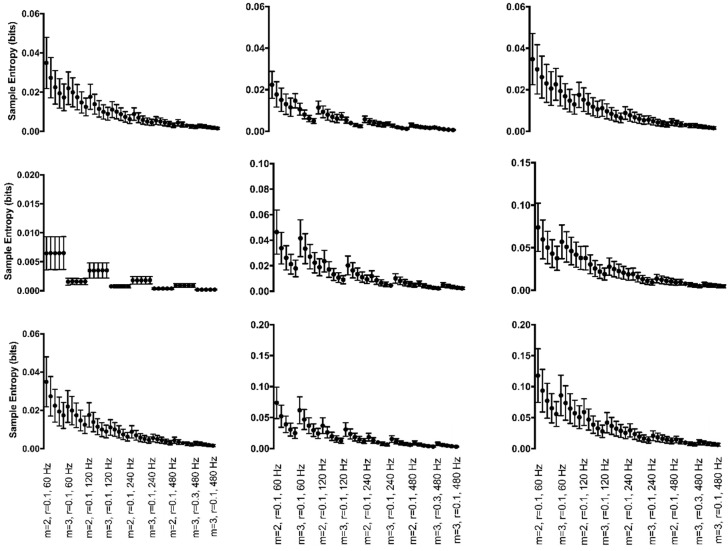
Mean sample entropy of continuous, generated time series with periodic (left column), chaotic (middle column), and random (right column) manipulations for each Hz*r*m parameter combination. Manipulations to cycle amplitude are shown in the top row, cycle period in the middle row, and both cycle amplitude and period in the bottom row. Sample entropy values are displayed on the y-axis whereas all parameter combinations are plotted along the x-axis starting with *m* = 2, *r* = 0.10, and 60 Hz at the left and moving to *m* = 3, *r* = 0.30, and 480 Hz at the right. For clarity, only the combination with *r* = 0.10 is labeled for each combination of *m* and sampling rate except the final combination.

When cycle amplitude was subjected to periodic manipulations, this led to a significant 3-way interaction between Hz*r*m (F(12,120) = 196.9, *p* < 0.001) (Figure 7, top row, left column). Periodic manipulations to cycle period led to a significant interaction between Hz*m (F(3,30) = 219.2, *p* < 0.001) (Figure 7, middle row, left column). When *m* increased from 2 to 3, irregularity increased at a greater rate as sampling rate increased from 60 Hz to 480 Hz. In addition, periodic manipulations to the combination of both cycle period and amplitude led to a significant 3-way interaction between Hz*r*m (F(12,120) = 196.9, *p* < 0.001) (Figure 7, bottom row, left column). Findings were similar for both cycle amplitude and the combination of cycle period and amplitude manipulations. Irregularity was always greater when *m* = 3 compared to *m* = 2 at all *r* values. Irregularity increased with the increase in sampling rate from 60 Hz to 480 Hz. An increase in the *r* value made little difference in irregularity when the sampling rate was lower but increased at a greater rate with increasing *r* at the greater sampling rates (e.g., 480 Hz). It was found that 597 of the possible 780 pairwise comparisons were significant for both manipulations.

#### 3.3.2. Chaotic Manipulations

Chaotic manipulations to cycle amplitude led to a significant 3-way interaction between Hz*m*r (F(12,120) = 272.6, *p* < 0.001) (Figure 7, top row, middle column). Irregularity increased as *r* increased across all Hz and *m*; the increase in irregularity was greater when *m* = 3 and as *r* increased. Irregularity increased when *m* increased from 2 to 3; however, the increase was greater when Hz was larger. Cycle period manipulations led to a significant 3-way interaction between Hz*r*m (F(12,120) = 227.1, *p* < 0.001) (Figure 7, middle row, middle column). The interaction between *m* and *r* was of particular interest. When *r* was set equal to 0.10 or 0.15, irregularity was greater with *m* = 3; however, there was no difference in irregularity between *m* of 2 and 3 when *r* = 0.20. As *r* increased to 0.25 and 0.30, irregularity was greater when *m* = 2 compared to *m* = 3. Irregularity was always greater as the sampling rate increased across both *m* values. An increase in the *r* value made little difference in irregularity when the sampling rate was lower but increased at a greater rate with increasing *r* at the greater sampling rates (e.g., 480 Hz). It was found that 595 of the possible 780 pairwise comparisons were significant. The combination of chaotic manipulations to both cycle period and amplitude led to a significant 3-way interaction between Hz*r*m (F(12,120) = 51.0, *p* < 0.001) (Figure 7, bottom row, middle column). Irregularity was greater when *m* = 3 compared to *m* = 2 at all *r* values. Irregularity was greater as the sampling rate increased across both *m* values. An increase in the *r* value made little difference in irregularity when the sampling rate was lower but increased at a greater rate with increasing *r* at the greater sampling rates (e.g., 480 Hz). It was found that 602 of the possible 780 pairwise comparisons were significant.

#### 3.3.3. Random Manipulations

Random manipulations to cycle amplitude resulted in a significant 3-way interaction between Hz*m*r (F(12,120) = 218.4, *p* < 0.001) (Figure 7, top row, right column). Irregularity increased as *r* increased across all Hz and *m*; the increase in irregularity was greater when *m* = 3 and as *r* increased. Irregularity increased when *m* increased from 2 to 3; however, the increase was greater when Hz was larger. Random manipulations to cycle period resulted in a significant 3-way interaction between Hz*r*m (F(12,120) = 215.8, *p* < 0.001) (Figure 7, middle row, right column). In addition, manipulations to cycle period plus amplitude resulted in a significant 3-way interaction between Hz*r*m (F(12,120) = 143.7, *p* < 0.001) (Figure 7, bottom row, right column). The findings were similar for both types of manipulations. Irregularity was always greater when *m* = 3 compared to *m* = 2 at all *r* values. When *m* increased from 2 to 3, the resulting irregularity increased at a greater rate as sampling rate increased from 60 Hz to 480 Hz. An increase in the *r* value made little difference in irregularity when the sampling rate was lower but increased at a greater rate with increasing *r* at the greater sampling rates (e.g., 480 Hz). 

### 3.4. Discrete, Generated Data

Periodic manipulations had the greatest effect on cycle amplitude (Table 4). Whereas, chaotic and random manipulations lead to a main effect of *m* for cycle amplitude and a main effect of *r* for cycle period.

#### 3.4.1. Periodic Manipulations

**Figure 8:**
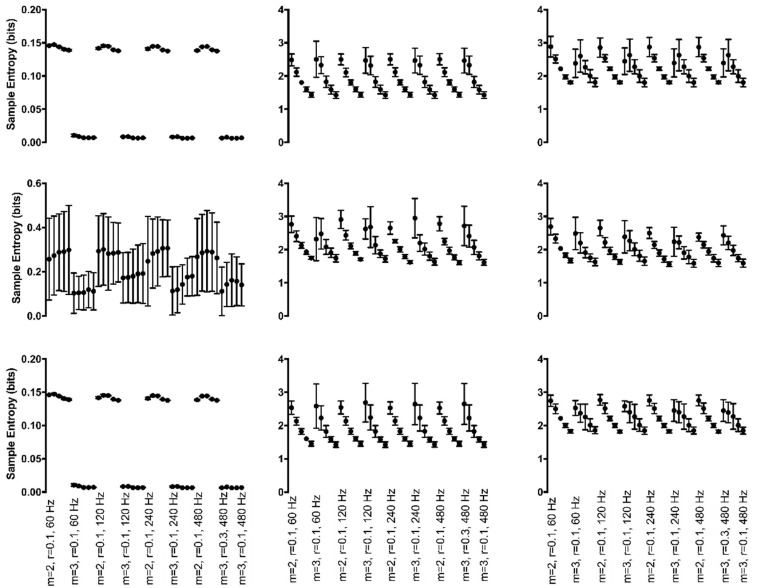
Mean sample entropy of discrete, generated time series with periodic (left column), chaotic (middle column), and random (right column) manipulations for each Hz*r*m parameter combination. Manipulations of the cycle amplitude are shown in the top row, cycle period in the middle row, and both cycle amplitude and period in the bottom row. Sample entropy values are displayed on the y-axis. All parameter combinations are plotted along the x-axis starting with *m* = 2, *r* = 0.10, and 60 Hz at the left and moving to *m* = 3, *r* = 0.30, and 480 Hz at the right. For clarity only the combination with *r* = 0.10 is labeled for each combination of m and sampling rate except the final combination. NOTE: y-axis scales were adjusted to best display data. They may not be the same across subfigures.

Discrete data with periodic manipulations to cycle amplitude resulted in a significant 3-way interaction between Hz*r*m (F(12,120) = 13.5, *p* < 0.001) (Figure 8, top row, left column). Irregularity was always greater when *m* = 2 as compared to when *m* = 3 across all values and sampling rates. Irregularity was dependent on the combination of sampling rate and the selected *r* value. Irregularity increased from *r* = 0.10 to *r* = 0.15; however, decreased as *r* increased from 0.15 to 0.30. The change in irregularity was dependent on the sampling rate at *r* values of 0.10 and 0.15 and did not have as much of an effect when *r* values greater or equal to 0.20. When subjected to cycle period manipulations, one main effect, the effect of *m*, was found (F(1,10) = 29.1, *p* < 0.001) (Figure 8, middle row, left column). Irregularity was always greater when *m* = 2 as compared to when *m* = 3. When both cycle period and amplitude manipulations were applied to the data, a significant 3-way interaction between Hz*r*m was found (F(12,120) = 15.8, *p* < 0.001) (Figure 8, bottom row, left column). Opposite of the manipulations independently, irregularity was always greater when *m* = 3 as compared to when *m* = 2 across all *r* values. Similar to amplitude manipulations alone, irregularity was dependent on the combination of sampling rate and the selected *r* value; however, in the opposite direction. Irregularity decreased from *r* = 0.10 to *r* = 0.15 and proceeded to increase as *r* increased from 0.15 to 0.20. When *r* was greater or equal to 0.25, irregularity was fairly consistent. The change in irregularity was dependent on the sampling rate at *r* values of 0.10 and 0.15 and little effect when *r* values greater or equal to 0.20.

#### 3.4.2. Chaotic Manipulations

Chaotic manipulations to cycle amplitude resulted in a main effect of *r* (F(4,16) =50.9, *p* < 0.001) (Figure 8, top row, middle column). Irregularity increased as *r* increased. The manipulations to cycle period resulted in a main effect of *r* (F(4,8) = 54.3, *p* < 0.001) (Figure 8, middle row, middle column). Regularity increased as the *r* value increased across both sampling rates and *m* values. The combination of cycle period and amplitude manipulations resulted in a main effect of *r* (F(4,36) = 88.8, *p* < 0.001) (Figure 8, bottom row, middle column). Regularity increased as the *r* value increased across both sampling rate and *m* values.

#### 3.4.3. Random Manipulations

Random manipulations to cycle amplitude also resulted in a significant main effect of *r* (F(1,4) = 173.5, *p* < 0.001) (Figure 8, top row, right column). Regularity significantly increased as *r* increased. Random manipulations to cycle period resulted in a significant 3-way interaction between Hz*r*m (F(12,60) = 2.2, *p* = 0.021) (Figure 8, middle row, right column). With increasing *r* values, regularity increased. Compared to *m* = 3, irregularity was always greater when *m* = 2. Irregularity was greater at *m* = 2 when the *r* value was smallest. Irregularity was dependent on the combination of sampling rate and the selected *m* value. As sampling rate increased, regularity increased when *m* = 2; however, when *m* = 3, 60 Hz and 480 Hz resulted in the same regularity with 120 Hz producing the greatest irregularity and 240 Hz producing the least irregularity. Combining manipulations to cycle period and amplitude resulted in a main effect of *r* (F(4,24) = 40.1, *p* < 0.001) (Figure 8, bottom row, right column). Regularity increased as the *r* value increased.

## 4. Discussion

**Figure 9:**
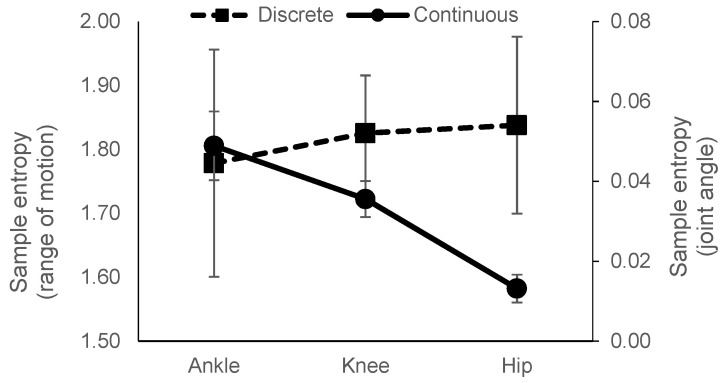
Representative results of sample entropy calculated from range of motion (left axis) and joint angle (right axis) at 120Hz, *m* = 2, *r* = 0.25. Note: Left and right axes have different scales.

As there has been a shift in the use of discrete data to continuous data types for use in sample entropy calculation, there is a need to understand how the use of data type will affect conclusions drawn. In particular, this paper aimed to examine the sample entropy between the three lower extremity joints while walking. Using both continuous (joint angle) and discrete (range of motion) data types, we aimed to understand if relative sample entropy between the joints would be similar for both data types. The relationship across the joints was not similar for joint angle and range of motion data. Using range of motion data, there was no difference in values across the three joints. However, the use of joint angle data produced sample entropy values that significantly decreased, an increase in regularity, from the ankle to the hip. In addition, sample entropy values calculated from joint angle data were not similar to those from range of motion data (Figure 5, Figure 6, and Figure 9). Range of motion data resulted in sample entropy values well above 1 yet, joint angle data resulted in values well below 1. Furthermore, in general, sample entropy calculated from joint angle data was more affected by changes in input parameters and sampling rate compared to sample entropy calculated from range of motion data. We observed that across the three joints, only changes in the input parameter *r* had a significant effect for range of motion data.

Previous work has suggested that the stable calculation of sample entropy requires 2000 data points [14]. It could be tempting to use continuous data series to achieve this number; however, it has not been proven that this is an appropriate input for the calculation of sample entropy, or that calculation of sample entropy from continuous data would lead to similar results as when calculated from discrete data. It was found that relationships between the joints and sample entropy values themselves were not similar between the two types of data sets, possibly due to the dynamics of the system that each capture. Discrete data captures a general picture of walking regularity and cycle-to-cycle regularity. However, continuous data may capture the dynamics of patterns occurring within each cycle. This may explain the significant differences between joint angles with the ankle being the most irregular and the hip angle as the most regular. During walking, changes in ankle angle are required during each stride to maintain balance during stance and for toe clearance during swing. At the knee, changes in joint angle are required to position the foot prior to stance. Whereas, changes in hip joint angle affect stride length and occur in a similar cyclical pattern from stride to stride. These differences in joint function may explain the relative changes in regularity using joint angle data. Further, lag values used in the calculation of sample entropy were smallest for the ankle, suggesting that changes in dynamics of the ankle joint occur more rapidly than the hip and knee. Data within the ankle joint angle is not as redundant as data within the knee and hip. It is possible that the ankle joint angle requires a higher sampling rate to capture the all of the dynamics of the joint as compared to the hip and knee.

Another explanation for the difference is likely due to the nature of the calculation of sample entropy itself. When sample entropy is calculated, the value *r* is multiplied by the standard deviation of the data series to set the tolerance used to determine if vectors of data points are counted as similar. When continuous data is used as the input, the difference between consecutive data points is much smaller relative to the standard deviation, compared to discrete data. This results in many more vectors of length *m* and *m* + 1 falling within the tolerance, counted as similar, and a much smaller sample entropy value. This is true even more so at higher sampling rates. Because of the greater number of vectors falling within the tolerance, changes in the value of *r* will have a greater effect on the actual tolerance relative to the differences between consecutive data points, and hence, on the number of data series falling within the tolerance. When *m* is increased, the number of data series of length *m*+1 that are counted similar at *m* will reduce, with the rate of reduction becoming greater for larger values of *m*. Future work should explore the effect on sample entropy when using values of *m* greater than 3 with continuous data. This could be of particular interest if the purpose of the investigation is to determine the regularity of longer patterns within a cycle. 

Early biological entropy studies focused on the use of discrete data sets. This could be due to the volatility of continuous data to changes in input parameters and sampling rate. In addition, the motivation for development of an algorithm to calculate entropy was to produce a measure that provided not only insight into the dynamics of the system, but also was not affected by noise in the system. It was stated that approximate entropy provided “de facto noise filtering and artifact insensitivity” [28], p.111, likely due to the nature of the data used in its calculation. In the current study, sample entropy calculated from continuous data was more affected by changes in both input parameters and sampling rates as compared to discrete data. Based on the results of this work, it is recommended that discrete data be used for the calculation of sample entropy. The effect of sampling rate on discrete data should not be completely discounted however, especially if the input parameter to the sample entropy calculation is interval (time) data. If data are collected at 60Hz the minimum interval between points is 0.0167 s. Should these data have a standard deviation of 0.1s, r values less than 0.167 will give a tolerance less than the minimum interval between data points and larger *r* values will give tolerances greater than the minimum interval. It is important, therefore, to consider the resolution (minimum interval between points) of the data as determined by the sampling frequency in conjunction with selection of *r* value for calculation of sample entropy.

If the entropy of the dynamics within a cycle are of interest, it is recommended that careful consideration be given to sampling rate and input parameters, as results could be due to a parameter artifact and not a true finding. In addition, it is recommended that a lag be used in the calculation of entropy with continuous data. Furthermore, if continuous data are used, it is crucial that input parameters and sampling frequency are carefully selected and consistent when comparisons are made between studies. Whereas, comparisons based on discrete data are less sensitive to methodological differences, except for the input parameter *r*. A very recent paper [39] investigated the use of filtered and downsampled data for the calculation of sample entropy from center of pressure data measured during treadmill walking. This work suggested that when data are subjected to filtering of the high frequency components and resampling to have the same number of average data points per stride sample entropy maintained the same directional differences across two walking conditions. The data in the current study were not filtered. It has been suggested that filtering will remove some of the inherent variability within the signal and that a consistent level of measurement noise will be present. Any differences can therefore be attributed to the differences in the signals [40,41]. It has also been shown that the sample entropy calculation is robust in the presence of noise [42]. Given the different data treatments it is difficult to draw conclusions when comparing the results this recent paper with the current study.

To further understand the influence of input parameters, sampling rate, and investigated signal type (continuous or discrete), the present study calculated sample entropy from generated continuous and discrete signals of a theoretically known type. These signals were based on a sine wave in which temporal and spatial periodicity were periodically, chaotically, and randomly manipulated by altering the cycle period, cycle amplitude, or both. In agreement with the biologically-derived data, sample entropy values calculated from generated, continuous data were not similar to those from generated, discrete data (see Figure 7 and Figure 8). In addition, continuous data resulted in sample entropy values well below 1 similar to the biological, joint angle data. However, this was not always true for the discrete data. Periodic manipulations to cycle amplitude, cycle period, and cycle period and amplitude resulted in values well below 1, similar to the continuous data. In addition, any manipulation of cycle amplitude resulted in sample entropy values well below 1. In general, it was observed that the generated, discrete-based signals were less sensitive to changes in input parameters or sampling rate compared to the continuous signals. This was primarily true when the signals were subjected to chaotic or random manipulations or only for the cycle period when subjected to periodic manipulations. Values of sample entropy calculated from generated signals were most similar to the values calculated from biological signals for data with chaotic and random fluctuations to cycle period and both cycle period and amplitude. This suggests that these types of signals, or a combination thereof, are more likely present in biological data than truly periodic fluctuations. 

There are several limitations to this study. The generated data used in this study may not properly reflect the types of biological signals from which sample entropy is calculated. While many biological signals are cyclical, their underlying characteristics may not be truly reflected by a sine wave. Further, the manipulations were applied individually and in combination with the cycle period and cycle amplitude. Biological signals will most likely show fluctuations in both period and amplitude; however, it is not known if the level of fluctuation is consistent with the manipulations applied to the generated data. Data recorded from biological signals generally includes some level of noise. The generated signals used in this study did not contain noise. Studies on the sample entropy values calculated from discrete and continuous data with known levels of noise present may provide further useful evidence concerning the information these data types provide. Signals containing multiple sine waves may provide a more realistic signal of high regularity for comparison of sample entropy values calculated from discrete and continuous data.

## 5. Conclusions

The calculation of sample entropy from continuous data provided results different to those determined when discrete data was used. The data types differed in terms of the relationship between joints and the sample entropy values themselves. Thus, results between the two data types cannot be compared. It is possible that calculation of sample entropy from continuous data may provide information about the regularity of fluctuations which occur within the cyclical movement, which cannot be captured from discrete data taken from these cycles. However, sample entropy calculated from continuous data was susceptible to the sampling rate of the data, as well as the parameters *m* and *r*. While calculation of sample entropy from discrete data was more robust to changes in sampling rate and *m*, it was affected by changes in *r*. It is recommended that discrete data be used for the calculation of sample entropy. However, if continuous data must be used, it is recommended that a lag be used and careful consideration be given to sampling rate and input parameters. The volatility of continuous data to input parameters and sampling rate may result in findings reported that are not true findings and are due to a parameter artifact. 

## Figures and Tables

**Figure 3:**
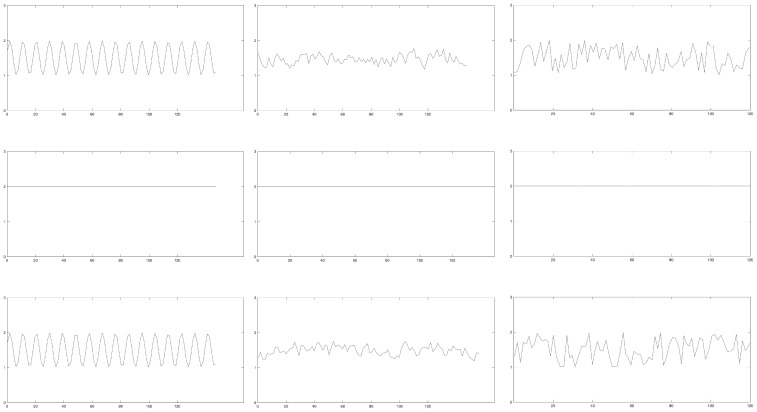
A 120-second segment of generated discrete data for periodic (**left**), chaotic (**middle**), and random (**bottom**) for amplitude (top), period (middle), and period + amplitude (bottom) manipulations.

**Table 1:** Mean (range) lag values used to calculate sample entropy on continuous time series.

	60 Hz	120 Hz	240 Hz	480 Hz
**Biological Data**				
Ankle Angle	8 (5–10)	16 (10–20)	31(20–39)	62 (40–78)
Knee Angle	10 (8–12)	18 (15–23)	36 (30–46)	72 (59–92)
Hip Angle	13 (11–17)	26 (22–33)	51(44–65)	102 (87–130)
**Generated Data**				
Periodic Manipulations	13 (6–17)	26 (12–34)	52 (24–68)	104 (48–136)
Chaotic Manipulations	13 (6–17)	26 (12–34)	52 (24–68)	104 (48–136)
Random Manipulations	13 (6–17)	26 (12–34)	52 (24–68)	104 (48–136)

Note: Values rounded to the nearest whole number.

**Table 2:** Statistical results for joint angle data (continuous data) and range of motion (discrete data) from all three lower extremity joints. Significant values are shown in bold type (*p* < 0.05). Joint angles (continuous) data were compared separately to range of motion data (discrete) data.

		Joint Angle		Range of Motion	
	Model	F-Value	*p*-Value	F-Value	*p*-Value
**Ankle**	Hz	294.7	**< 0.0001**	0.68	0.58
	*m*	255.5	**< 0.0001**	0.57	0.49
	*r*	105.9	**< 0.0001**	136.9	**< 0.0001**
	Hz*m	249.6	**< 0.0001**	1.1	0.37
	Hz*r	114.3	**< 0.0001**	1.1	0.39
	m*r	7.2	**< 0.0001**	0.46	0.77
	Hz*m*r	6.9	**< 0.0001**	1.0	0.43
**Knee**	Hz	481.8	**< 0.0001**	8.3	**0.02**
	*m*	84.0	**< 0.0001**	0.01	0.92
	*r*	35.1	**< 0.0001**	42.5	**< 0.0001**
	Hz*m	86.7	**< 0.0001**	1.1	0.40
	Hz*r	38.4	**< 0.0001**	2.3	**0.04**
	m*r	7.9	**< 0.0001**	1.4	0.33
	Hz*m*r	8.9	**< 0.0001**	0.40	0.95
**Hip**	Hz	204.9	**< 0.0001**	0.10	0.96
	*m*	188.9	**< 0.0001**	0.41	0.57
	*r*	40.0	**< 0.0001**	37.9	**< 0.0001**
	Hz*m	193.0	**< 0.0001**	1.5	0.28
	Hz*r	31.0	**< 0.0001**	1.9	0.07
	m*r	1.6	0.20	0.22	0.92
	Hz*m*r	1.7	0.09	4.7	**< 0.0001**

**Table 3:** Statistical results for continuous, generated data subjected to periodic, chaotic, and random manipulations to the sinusoids’ cycle amplitude, cycle period, and both cycle amplitude and period combined. Significant values are shown in bold (*p* < 0.05).

		Periodic Manipulation		Chaotic Manipulation		Random Manipulation	
	Model	F-Value	*p*-Value	F-Value	*p*-Value	F-Value	*p*-Value
**Cycle amplitude**	Hz	40.5	**< 0.0001**	273.0	**< 0.0001**	220.3	**< 0.0001**
	*m*	16921.0	**< 0.0001**	308.9	**< 0.0001**	207.7	**< 0.0001**
	*r*	140.9	**< 0.0001**	249.1	**< 0.0001**	224.5	**< 0.0001**
	Hz*m	40.2	**< 0.0001**	296.6	**< 0.0001**	217.9	**< 0.0001**
	Hz*r	16.0	**< 0.0001**	252.9	**< 0.0001**	226.2	**< 0.0001**
	m*r	140.7	**< 0.0001**	287.7	**< 0.0001**	206.7	**< 0.0001**
	Hz*m*r	15.8	**< 0.0001**	272.6	**< 0.0001**	218.4	**< 0.0001**
**Cycle period**	Hz	225.7	**< 0.0001**	211.1	**< 0.0001**	219.7	**< 0.0001**
	*m*	201.6	**< 0.0001**	19.2	**0.001**	157.1	**< 0.0001**
	*r*	1.4	0.27	204.1	**< 0.0001**	217.1	**< 0.0001**
	Hz*m	219.2	**< 0.0001**	30.6	**< 0.0001**	158.7	**< 0.0001**
	Hz*r	0.88	0.57	204.0	**< 0.0001**	217.0	**< 0.0001**
	m*r	1.1	0.38	226.4	**< 0.0001**	217.1	**< 0.0001**
	Hz*m*r	0.90	0.55	227.1	**< 0.0001**	215.8	**< 0.0001**
**Cycle period and amplitude**	Hz	225.4	**< 0.0001**	266.2	**< 0.0001**	225.7	**< 0.0001**
	*m*	219.8	**< 0.0001**	124.9	**< 0.0001**	185.9	**< 0.0001**
	*r*	224.9	**< 0.0001**	273.7	**< 0.0001**	245.5	**< 0.0001**
	Hz*m	221.2	**< 0.0001**	123.9	**< 0.0001**	186.6	**< 0.0001**
	Hz*r	223.1	**< 0.0001**	273.2	**< 0.0001**	245.8	**< 0.0001**
	m*r	195.9	**< 0.0001**	67.3	**< 0.0001**	143.5	**< 0.0001**
	Hz*m*r	196.9	**< 0.0001**	51.0	**< 0.0001**	143.7	**< 0.0001**

**Note.** Periodic, chaotic, and random refer to the type of manipulations applied to the signal. Cycle period, cycle amplitude, and cycle period and amplitude refer to the features of the signal which were manipulated.

**Table 4:** F- and *p*-values for periodic, chaotic, and random manipulations to the sinusoids’ cycle amplitude, cycle period, and both cycle amplitude and period combined on discrete, generated data. Significant values are shown in bold (*p* < 0.05).

		Periodic		Chaotic		Random	
	Model	F-Value	*p*-Value	F-Value	*p*-Value	F-Value	*p*-Value
**Cycle amplitude**	Hz	45.1	**< 0.0001**	0.16	0.92	0.58	0.64
	*m*	5472795	**< 0.0001**	0.47	0.51	0.71	0.45
	*r*	246.1	**< 0.0001**	236.3	**< 0.0001**	50.9	**< 0.0001**
	Hz*m	25.8	**< 0.0001**	0.69	0.57	0.31	0.82
	Hz*r	25.6	**< 0.0001**	0.17	1.0	0.41	0.96
	m*r	883.2	**< 0.0001**	2.5	0.06	1.6	0.21
	Hz*m*r	13.5	**< 0.0001**	0.57	0.86	1.0	0.43
**Cycle period**	Hz	0.38	0.77	0.83	0.53	0.56	0.65
	*m*	29.1	**< 0.0001**	0.09	0.79	17.5	**0.009**
	*r*	1.8	0.15	54.3	**< 0.0001**	81.2	**< 0.0001**
	Hz*m	1.9	0.16	1.1	0.41	3.7	**0.04**
	Hz*r	0.87	0.58	0.86	0.59	1.3	0.22
	m*r	1.4	0.24	0.10	0.98	5.4	**0.004**
	Hz*m*r	0.78	0.67	1.1	0.39	2.2	**0.02**
**Cycle period and amplitude**	Hz	45.1	**< 0.0001**	1.5	0.23	0.70	0.56
	*m*	5472795	**< 0.0001**	0.44	0.53	0.02	0.90
	*r*	246.1	**< 0.0001**	88.8	**< 0.0001**	40.1	**< 0.0001**
	Hz*m	25.8	**< 0.0001**	0.55	0.65	0.52	0.67
	Hz*r	25.6	**< 0.0001**	1.1	0.40	0.94	0.51
	m*r	883.2	**< 0.0001**	0.54	0.71	2.7	0.06
	Hz*m*r	13.5	**< 0.0001**	1.5	0.13	0.88	0.57

**Note.** Periodic, chaotic, and random refer to the type of manipulations applied to the signal. Cycle period, cycle amplitude, and cycle period and amplitude refer to the features of the signal which were manipulated.

## Supplementary Materials

Software S.1: Custom Matlab ® codes, available at the University of Nebraska at Omaha’s Digital Commons.
